# Relationship Between Baseline Rectal Tumor Length and Magnetic Resonance Tumor Regression Grade Response to Chemoradiotherapy: A Subanalysis of the TRIGGER Feasibility Study

**DOI:** 10.1245/s10434-022-11914-5

**Published:** 2022-06-30

**Authors:** Nicola Hodges, Nicholas Battersby, Sheela Rao, Gina Brown, the TRIGGER Study Group, Gayathri Anandappa, David Cunningham, Diana Tait, Paris Tekkis, Irene Chong, Katharine Aitken, Ian Chau, Shahnawaz Rasheed, Svetlana Balyasnikova, Brendan Moran, Stephen Falk, Bruce Sizer, Graham Branagan, Lorcan O’Toole, Madhavi Adusumalli, Iris Nagtegaal, Katharina Von Loga, Andrew Thrower, Andrew Jackson, Huw Roach, Hussein Hassan, Michael Carss, Andrew Bateman, Mark Wills, Caroline Martin, Ceri Evans, Emily Robinson, Zohra Zenasni, Michelle Frost, Karen Thomas, Francesco Di Fabio, Rayesh Rawlani, Hayley Cousins, Rachel Thomas, Jessica Jenkins, Thomas Strawson-Smith, Axel Walther, Timothy Spencer, Tim Robinson, Elysia Gower, Newton Wong, Sharon Short, Jennifer Collins, Celine Driscoll, Louies Mabelin, Georgios Bozas, Elaine Heeney, Mohammad Hegab, Lehentha Mattocks, Nick West, Phil Quirke, Kil Yeon Lee, Tania Rodrigues, Art Hiranyakai, Rodney Lynch, Bawantha Gamage

**Affiliations:** 1 The Royal Marsden NHS Foundation Trust and Imperial College London, Sutton, UK; 2 Imperial College, London, UK; 3 Royal Cornwall Hospital, Truro, UK; 4 The Royal Marsden NHS Foundation Trust, Sutton, UK; 5 Basingstoke and North Hampshire Hospital, Basingstoke, UK; 6 University Hospitals Bristol NHS Foundation Trust, Bristol, UK; 7 East Suffolk and North Essex NHS Foundation Trust, Colchester, UK; 8 Salisbury NHS Foundation Trust, Salisbury, UK; 9 Diana, Princess of Wales Hospital, Norther Lincolnshire and Goole Hospitals NHS Foundation Trust, Grimsby, UK; 10 North Tees and Hartlepool NHS Foundation Trust, Hartlepool, UK; 11 Radboud University Medical Centre, Nijmegen, The Netherlands; 12 Leeds Teaching Hospitals NHS Trust, Salisbury, UK; 13 Kyung Hee University Medical Centre, Seoul, South Korea; 14 Hospital da Luz, Lisbon, Portugal; 15 Bangkok Hospital, Bangkok, Thailand; 16 Barwon Health, University of Geelong, Geelong, Australia; 17 Colombo South Teaching Hospital, University Surgical Unit, University of Sri Jayewardenepura, Jayewardenepura, Sri Lanka

## Abstract

**Background:**

It is widely believed that small rectal tumors are more likely to have a good response to neoadjuvant treatment, which may influence the selection of patients for a ‘watch and wait’ strategy.

**Objective:**

The aim of this study was to investigate whether there is a relationship between baseline tumor length on magnetic resonance imaging (MRI) and response to chemoradiotherapy.

**Method:**

The 96 patients with locally advanced rectal cancer randomised (2:1–intervention:control) in the TRIGGER feasibility study where eligible. Baseline tumor length was defined as the maximal cranio-caudal length on baseline MRI (mm) and was recorded prospectively at study registration. Magnetic resonance tumor regression grade (mrTRG) assessment was performed on the post-chemoradiotherapy (CRT) MRI 4–6 weeks (no later than 10 weeks) post completion of CRT. This was routinely reported for patients in the intervention (mrTRG-directed management) arm and reported for the purposes of this study by the central radiologist in the control arm patients. Those with an mrTRG I/II response were defined as ‘good responders’ and those with an mrTRG III–V response were defined as ‘poor responders’.

**Results:**

Overall, 94 patients had a post-CRT MRI performed and were included. Forty-three (46%) patients had a good response (mrTRG I/II) and 51 (54%) patients had a poor response (mrTRG III/IV). The median tumor length of good responders was 43 mm versus 50 mm (*p* < 0.001), with considerable overlap in tumor lengths between groups.

**Conclusion:**

Baseline tumor length on MRI is not a clinically useful biomarker to predict mrTRG tumor response to CRT and therefore patient suitability for a deferral of surgery trial.

Neoadjuvant chemoradiotherapy (CRT) prior to high-quality total mesorectal excision is current standard of care in the management of high-risk locally advanced rectal cancer.^1^ Although CRT is recognized as reducing local recurrence rates, an individual’s tumor response to CRT is variable. It is now well-established that patients who have a complete pathological response to CRT have improved long-term survival compared with poor responders.^2–4^ This has led to the investigation of potential biomarkers to predict a patient’s or tumor’s response to CRT. However, such a biomarker remains elusive. Baseline rectal tumor characteristics have also been investigated to see whether they are useful in predicting response. Tumor length (size) and tumor height from the anal verge are two such markers. Tumor length, in particular, has been shown in a number of previous studies to correlate with response to CRT. The findings suggest the smaller the tumor length/size, the more favorable the response to CRT.^5–11^ This has helped clinicians justify the use of CRT in patients with smaller rectal cancers who may otherwise go straight to surgery in the hope they may sustain a complete response. Studies demonstrating a relationship between tumor length and response to CRT have not always determined an optimal size cut-off to determine those tumors more likely to respond to CRT.^5–7,10^ Other studies proposing length cut-offs have differed in their proposed cut-off value, with 4 cm,^11^ 5 cm,^9,12^ 6 cm,^8^ and 7 cm^13^ all proposed. This variation has translated into arbitrary length/size inclusion criteria in trials offering organ preservation as a management option, with cut-offs of 3 cm^14^ and 4 cm^15,16^ both used in the belief that larger tumors are unlikely to achieve a good response to CRT.

The TRIGGER trial is a multicenter, randomized control trial using the magnetic resonance tumor regression grade (mrTRG) to prospectively stratify the management of patients with locally advanced rectal cancer. mrTRG has previously been validated as a prognostic biomarker, with good responders (mrTRG I/II) having a 5-year overall survival of 72% versus a 27% 5-year overall survival for poor responders (mrTRG III/IV).^17^

The aim of this study was to perform a secondary analysis of patients randomized within the TRIGGER feasibility study to assess the relationship between baseline rectal tumor length and height from the anal verge with response to CRT as defined by mrTRG.

## Methods

The feasibility component of the TRIGGER trial included the first 96 randomized patients. Patients were eligible if they had locally advanced rectal cancer (defined on magnetic resonance imaging [MRI] as within 15 cm of the anal verge or within the rectum below the sigmoid take-off on MRI, and one or more of magnetic resonance circumferential resection margin [mrCRM] unsafe, ≥mrT3c, magnetic resonance extramural vascular invasion [mrEMVI] positive or N1c) confirmed with adenocarcinoma on biopsy and treated with long-course CRT (45–55 Gy). The TRIGGER feasibility protocol is available online and the trial was registered at ClinicalTrials.gov (identifier: NCT02704520).^18^

Tumor length was defined as the maximal cranio-caudal length measured on baseline MRI (in mm), and the tumor height was defined as the height from the anal verge on baseline MRI (in mm) and recorded at baseline registration. A specialist gastrointestinal radiologist at each of the participating seven UK centres performed the measurements on sagittal images. In the case of a long, curved tumor, broken straight lines were used to calculate the tumor length. No rectal filling was performed.

Patients were randomized 2:1 (intervention:control). All patients remaining within the trial post-CRT had a post-CRT MRI scan performed 4–6 weeks (no later than 10 weeks) following completion of CRT. mrTRG was reported for those patients in the intervention group and formed the basis of further management (deferral of surgery for good responders and consolidation chemotherapy for poor responders). Patients in the control group did not have mrTRG reported on their post-CRT MRIs. For the purpose of this study, the control group post-CRT MRI scans were read by the central reviewing radiologist and mrTRG was reported to enable this analysis. A description of the mrTRG is outlined in Table 1. Good responders were defined as mrTRG I/II and poor responders were defined as mrTRG III/IV.

**Table 1:** Magnetic resonance tumor regression grade

Good response	mrTRG I	Complete radiological response (linear scar only)
	mrTRG II	Good response (dense fibrosis, no obvious tumor signal)
Poor response	mrTRG III	Moderate response (>50% fibrosis and visible intermediate signal)
	mrTRG IV	Slight response (mostly tumor)
	mrTRG V	No response/regrowth of tumor

*mrTRG* magnetic resonance tumor regression grade

The potential predictive relationship between tumor length and height from the anal verge (as measured on baseline pre-CRT MRI) with mrTRG response on post-CRT MRI was assessed using the the Mann–Whitney U test and the independent samples median test. Statistical analysis was performed using IBM SPSS Statistics for Windows, version 28.0 (IBM Corporation, Armonk, NY, USA).

Ethics approval for the TRIGGER trial was obtained from the London-Surrey Borders Research Ethics Committee on 18 December 2015 (IRAS ID 156408).

## Results

**Fig. 1:**
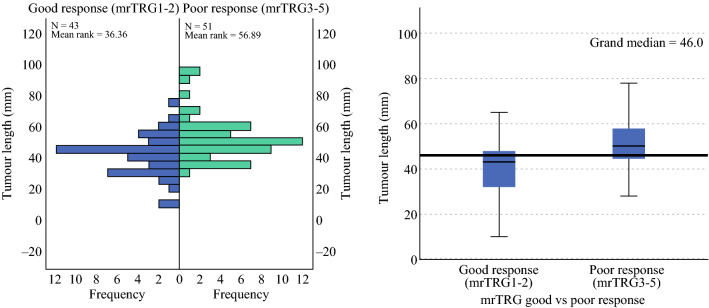
Tumor length (mm) on baseline magnetic resonance imaging versus mrTRG response to chemoradiotherapy. *mrTRG* magnetic resonance tumor regression grade

Overall, 96 patients were included in the TRIGGER feasibility study (registered between March 2016 and March 2019), 94 of whom had a post-CRT MRI performed and were thus included in this analysis. Baseline patient and tumor characteristics of the included patients are shown in Table 2. A good response to CRT (mrTRG I/II) was achieved in 43 patients (46%), while 51 patients (54%) were deemed to have had a poor response to CRT (mrTRG III/IV). Median tumor length (range) in those with a good response was 43 mm (10–73 mm), and 50 mm (28–96 mm) [*p* < 0.001] in those patients with a poor response (Fig. 1). Although the relationship between tumor length and mrTRG-assessed response to CRT was statistically significant (*p* ≤ 0.001), there was considerable overlap in tumor length between those who had a good response to CRT and those with a poor response (Fig. 1).

**Table 2:** Patient characteristics on baseline magnetic resonance imaging (pre-CRT) [*n* = 94]

	*N* (%)
*Age group, years*	
<70	58 (62)
≥70	36 (38)
*Sex*	
Male	71 (76)
Female	23 (24)
*BMI*^a^	
<18.5	1 (1)
18.5–24.9	34 (36)
25–29.9	35 (37)
30–39.9	16 (17)
≥40	4 (4)
*WHO performance status*	
0	70 (74)
1	24 (26)
2	0
*Radiological T-stage*	
mrT1	0
mrT2	8 (9)
mrT3a	5 (5)
mrT3b	22 (23)
mrT3c	39 (41)
mrT3d	10 (11)
mrT4 visceral	5 (5)
mrT4 peritoneal	5 (5)
*Radiological N-stage*	
mrN0	15 (16)
mrNode-positive	16 (17)
mrN1c	63 (67)
*mrCRM*	
Safe	15 (16)
Unsafe	79 (84)
*mrEMVI*	
Negative	24 (26)
Positive	70 (74)

*CRT* chemoradiotherapy, *BMI* body mass index, *mr* magnetic resonance, *mrCRM* magnetic resonance circumferential resection margin, *mrEMVI* magnetic resonance extramural vascular invasion

^a^BMI data for four patients were unavailable

Table 3 illustrates the percentage of ‘good responders’ who would have been excluded from this trial had tumor length cut-off values proposed in the literature been applied to this study; 35 of 43 (81%) good responders would have been ineligible if a tumor length of <30 mm had been used, 27 of 43 (63%) would have been ineligible with a cut-off of <40 mm, and 10 of 43 (23%) would have been ineligible had a cut-off of <50 mm been imposed.

**Table 3:** Percentage of good responders included if tumor length ‘cut-off’ values applied to the inclusion criteria

Proposed ‘cut-off’ length (mm)	Good responders [*n* = 43]	
	Included [*n* (%)]	Excluded [*n* (%)]
<30	8 (19)	35 (81)
<40	16 (37)	27 (63)
<50	33 (77)	10 (23)
<60	41 (95)	2 (5)
<70	42 (98)	1 (2)

**Fig. 2:**
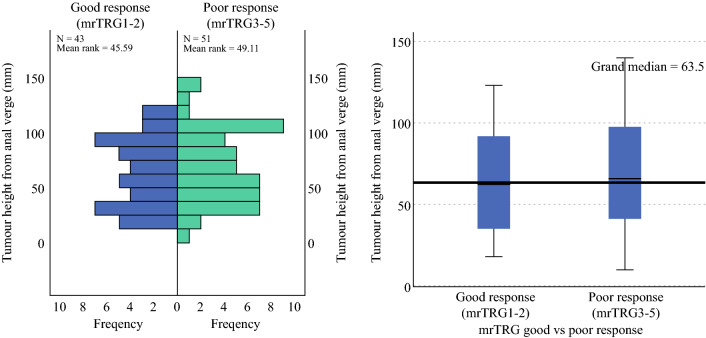
Tumor height from the anal verge (mm) versus mrTRG response to chemoradiotherapy. *mrTRG* magnetic resonance tumor regression grade

The median tumor height (range) from the anal verge in those with a good response was 63 mm (18–123 mm) and 66 mm (10–140 mm) in those with a poor response (*p* = 0.836) (Fig. 2).

## Discussion

Previous studies investigating the relationship between baseline tumor size/length and height from the anal verge have been hampered by their retrospective nature, variable tools used to measure tumor length and height from the anal verge (rigid/flexible sigmoidoscopy, endorectal ultrasound, MRI), and variable measures of response to CRT (clinical complete response, pathologic tumor regression grade, and pathological complete response). The TRIGGER trial provides a unique opportunity to investigate these baseline tumor characteristics in a clearly defined population of patients with locally advanced rectal cancer. Using data from a prospective trial, with a standardized CRT regimen, standardized baseline measurements, and standardized measure of response, the usefulness of these locally advanced rectal cancer tumor characteristics as predictors of response to CRT, and their suitability for deferral of surgery, can be more meaningfully assessed.

The magnetic resonance tumour regression grade (mrTRG) on the post-CRT MRI was used as the endpoint to assess response to CRT in this analysis. This is arguably a more clinically relevant endpoint than the pathologic tumour regression grades or pathological complete response rates used as endpoints in other studies. Similarly to pathologic assessment of response to CRT, mrTRG has been validated as a prognostic biomarker in locally advanced rectal cancer patients undergoing neoadjuvant therapy for rectal cancer.^17^ Pathologic assessments of response to CRT however have the disadvantage of only being able to be assessed following surgical resection of the tumour and are therefore unhelpful in determining factors which may predict patients suitable for organ preservation or a deferral of surgery pathway. Furthermore, pathological complete response to CRT is recognised as being time-dependent.^19–21^ Finding tumour cells within a resection specimen at the time of surgical excision does not provide information as to whether further tumour regression would have occurred had a longer time interval passed between the completion of CRT and surgery, nor does it provide information regarding the viability of those tumour cells and whether or not they have in fact been sterilised.

Similar to previous studies, this secondary analysis of the TRIGGER feasibility study, has confirmed a statistically significant relationship between a smaller tumour size at baseline and greater response to CRT.^5,6,9,13,22^ Although statistically significant, a difference of 7mm in the median size between good responders and poor responders cannot be classified as clinically significant. This is concordant with the findings of Wallin et al who concluded that the difference in tumour size between responders and non-responders to CRT was too small to be useful as a baseline predictive marker.^23^ More recently Jankowski et al concluded that those patients with a tumour ≥7cm in size were of low likelihood to achieve a complete clinical response to CRT and thus be suitable for deferral of surgery.^13^ We have also shown that if tumour-length cut-off values used in other organ preservation studies had been used as part of the inclusion criteria for the TRIGGER trial, between 23 and 81% of patients suitable for deferral of surgery would have been excluded at the outset.

Our finding of no significant relationship between tumour height on baseline MRI and mrTRG response to CRT is consistent with other recently published studies contradicting previous work that had suggested an inverse relationship between height from anal verge and response to CRT.^24,25^

Potential limitations of this analysis include the small number of patients (94) included and the fact that the TRIGGER trial only recruits patients with locally advanced rectal cancer. By definition this may lend itself to bias with inclusion of larger and lower rectal tumours. Contrary to this, the findings are generalisable to all patients with locally advanced rectal cancer currently recommended to be treated with neoadjuvant CRT as per the current ESMO guidelines.^1^ The protocol required the post CRT MRI be performed 4-6 weeks (no later than 10 weeks) following the completion of CRT. It is therefore possible the downstaging effects of radiotherapy may have still been ongoing at the time of the post-CRT MRI. Although this is a potential limitation of this study, it is likely the ongoing effects of radiotherapy would have had more impact on tumours that were larger at baseline, so if anything would strengthen the conclusion of our study rather than limit the generalizability.

## Conclusion

We have found no clinically relevant relationship between tumour length and tumour height from anal verge and response to neoadjuvant CRT in patients with locally advanced rectal cancer. Further research with the TRIGGER translational study is necessary to try and identify biomarkers at diagnosis capable of predicting a patient with LARC’s suitability for a deferral of surgery pathway following CRT.

## Appendix

### TRIGGER Study Group

**Table:**

First name	Last name	Institution
Gayathri	Anandappa	The Royal Marsden NHS Foundation Trust, Sutton, UK
David	Cunningham	The Royal Marsden NHS Foundation Trust, Sutton, UK
Diana	Tait	The Royal Marsden NHS Foundation Trust, Sutton, UK
Paris	Tekkis	The Royal Marsden NHS Foundation Trust, Sutton, UK
Irene	Chong	The Royal Marsden NHS Foundation Trust, Sutton, UK
Katharine	Aitken	The Royal Marsden NHS Foundation Trust, Sutton, UK
Ian	Chau	The Royal Marsden NHS Foundation Trust, Sutton, UK
Shahnawaz	Rasheed	The Royal Marsden NHS Foundation Trust, Sutton, UK
Svetlana	Balyasnikova	The Royal Marsden NHS Foundation Trust, Sutton, UK
Brendan	Moran	Basingstoke and North Hampshire Hospital, Basingstoke, UK
Stephen	Falk	University Hospitals Bristol NHS Foundation Trust, Bristol, UK
Bruce	Sizer	East Suffolk and North Essex NHS Foundation Trust, Colchester, UK
Graham	Branagan	Salisbury NHS Foundation Trust, Salisbury, UK
Lorcan	O’Toole	Diana, Princess of Wales Hospital, Norther Lincolnshire and Goole Hospitals NHS Foundation Trust, UK
Madhavi	Adusumalli	North Tees and Hartlepool NHS Foundation Trust, UK
Iris	Nagtegaal	Radboud University Medical Centre, Nijmegen, The Netherlands
Katharina	Von Loga	The Royal Marsden NHS Foundation Trust, Sutton, UK
Andrew	Thrower	Basingstoke and North Hampshire Hospital, Basingstoke, UK
Andrew	Jackson	Basingstoke and North Hampshire Hospital, Basingstoke, UK
Huw	Roach	University Hospitals Bristol NHS Foundation Trust, Bristol, UK
Hussein	Hassan	Diana, Princess of Wales Hospital, Norther Lincolnshire and Goole Hospitals NHS Foundation Trust, UK
Michael	Carss	North Tees and Hartlepool NHS Foundation Trust, UK
Andrew	Bateman	Salisbury NHS Foundation Trust, Salisbury, UK
Mark	Wills	Salisbury NHS Foundation Trust, Salisbury, UK
Caroline	Martin	The Royal Marsden NHS Foundation Trust, Sutton, UK
Ceri	Evans	The Royal Marsden NHS Foundation Trust, Sutton, UK
Emily	Robinson	The Royal Marsden NHS Foundation Trust, Sutton, UK
Zohra	Zenasni	The Royal Marsden NHS Foundation Trust, Sutton, UK
Michelle	Frost	The Royal Marsden NHS Foundation Trust, Sutton, UK
Karen	Thomas	The Royal Marsden NHS Foundation Trust, Sutton, UK
Francesco	Di Fabio	Basingstoke and North Hampshire Hospital, Basingstoke, UK
Rayesh	Rawlani	Basingstoke and North Hampshire Hospital, Basingstoke, UK
Hayley	Cousins	Basingstoke and North Hampshire Hospital, Basingstoke, UK
Rachel	Thomas	Basingstoke and North Hampshire Hospital, Basingstoke, UK
Jessica	Jenkins	University Hospitals Bristol NHS Foundation Trust, Bristol, UK
Thomas	Strawson-Smith	University Hospitals Bristol NHS Foundation Trust, Bristol, UK
Axel	Walther	University Hospitals Bristol NHS Foundation Trust, Bristol, UK
Timothy	Spencer	University Hospitals Bristol NHS Foundation Trust, Bristol, UK
Tim	Robinson	University Hospitals Bristol NHS Foundation Trust, Bristol, UK
Elysia	Gower	University Hospitals Bristol NHS Foundation Trust, Bristol, UK
Newton	Wong	University Hospitals Bristol NHS Foundation Trust, Bristol, UK
Sharon	Short	University Hospitals Bristol NHS Foundation Trust, Bristol, UK
Jennifer	Collins	East Suffolk and North Essex NHS Foundation Trust, Colchester, UK
Celine	Driscoll	East Suffolk and North Essex NHS Foundation Trust, Colchester, UK
Louies	Mabelin	East Suffolk and North Essex NHS Foundation Trust, Colchester, UK
Georgios	Bozas	Diana, Princess of Wales Hospital, Northern Lincolnshire and Goole Hospitals NHS Foundation Trust, UK
Elaine	Heeney	Diana, Princess of Wales Hospital, Northern Lincolnshire and Goole Hospitals NHS Foundation Trust, UK
Mohammad	Hegab	North Tees and Hartlepool NHS Foundation Trust, UK
Lehentha	Mattocks	Salisbury NHS Foundation Trust, Salisbury, UK
Nick	West	Leeds Teaching Hospitals NHS Trust
Phil	Quirke	Leeds Teaching Hospitals NHS Trust
Kil Yeon	Lee	Kyung Hee University Medical Centre, Seoul, South Korea
Tania	Rodrigues	Hospital da Luz, Lisbon, Portugal
Art	Hiranyakai	Bangkok Hospital, Bangkok, Thailand
Rodney	Lynch	Barwon Health, University of Geelong, Australia
Bawantha	Gamage	University Surgical Unit, University of Sri Jayewardenepura, Colombo South Teaching Hospital, Sri Lanka

## References

[1] Glynne-Jones R, Wyrwicz L, Tiret E (2017). Rectal cancer: ESMO Clinical Practice Guidelines for diagnosis, treatment and follow-up. Ann Oncol.

[2] Habr-Gama A, Perez RO, Nadalin W (2004). Operative versus nonoperative treatment for stage 0 distal rectal cancer following chemoradiation therapy: long-term results. Ann Surg.

[3] Capirci C, Valentini V, Cionini L (2008). Prognostic value of pathologic complete response after neoadjuvant therapy in locally advanced rectal cancer: long-term analysis of 566 ypCR patients. Int J Radiat Oncol Biol Phys.

[4] Martens MH, Maas M, Heijnen LA (2016). Long-term outcome of an organ preservation program after neoadjuvant treatment for rectal cancer. J Natl Cancer Inst.

[5] Garland ML, Vather R, Bunkley N, Pearse M, Bissett IP (2014). Clinical tumour size and nodal status predict pathologic complete response following neoadjuvant chemoradiotherapy for rectal cancer. Int J Colorectal Dis.

[6] Hammarström K, Imam I, Mezheyeuski A, Ekström J, Sjöblom T, Glimelius B (2021). A comprehensive evaluation of associations between routinely collected staging information and the response to (Chemo)radiotherapy in rectal cancer. Cancers (Basel).

[7] Fischer J, Eglinton TW, Frizelle FA (2021). Clinical predictors of response to chemoradiotherapy for rectal cancer as an aid to organ preservation. ANZ J Surg.

[8] Hajer J, Rim A, Ghorbel A (2021). Predictive factors associated with complete pathological response after neoadjuvant treatment for rectal cancer. Cancer/Radiotherapie.

[9] De Felice F, Izzo L, Musio D (2016). Clinical predictive factors of pathologic complete response in locally advanced rectal cancer. Oncotarget.

[10] Huh JW, Kim HR, Kim YJ (2013). Clinical prediction of pathological complete response after preoperative chemoradiotherapy for rectal cancer. Dis Colon Rectum.

[11] Park CH, Kim HC, Cho YB (2011). Predicting tumor response after preoperative chemo radiation using clinical parameters in rectal cancer. World J Gastroenterol.

[12] Janjan N, Khoo V, Abbruzzese Je, Al E (1999). Tumor downstaging and sphincter preservation with preoperative chemoradiation in locally advanced rectal cancer: the MD Anderson Cancer Center experience. Int J Radiat Oncol Biol Phys.

[13] Jankowski M, Pietrzak L, Rupiński M (2021). Watch-and-wait strategy in rectal cancer: is there a tumour size limit? Results from two pooled prospective studies. Radiother Oncol.

[14] Bach SP, Gilbert A, Brock K (2021). Radical surgery versus organ preservation via short-course radiotherapy followed by transanal endoscopic microsurgery for early-stage rectal cancer (TREC): a randomised, open-label feasibility study. Lancet Gastroenterol Hepatol.

[15] Rombouts AJM, Al-Najami I, Abbott NL (2017). Can we S ave the rectum by watchful waiting or T rans A nal microsurgery following (chemo) R adiotherapy versus T otal mesorectal excision for early RE ctal C ancer (STAR-TREC study)? Protocol for a multicentre, randomised feasibility study. BMJ Open.

[16] Rullier E, Vendrely V, Asselineau J (2020). Organ preservation with chemoradiotherapy plus local excision for rectal cancer: 5-year results of the GRECCAR 2 randomised trial. Lancet Gastroenterol Hepatol.

[17] Patel UB, Taylor F, Blomqvist L (2011). Magnetic resonance imaging-detected tumor response for locally advanced rectal cancer predicts survival outcomes: MERCURY experience. J Clin Oncol.

[18] Battersby NJ, Dattani M, Rao S (2017). A rectal cancer feasibility study with an embedded phase III trial design assessing magnetic resonance tumour regression grade (mrTRG) as a novel biomarker to stratify management by good and poor response to chemoradiotherapy (TRIGGER): Study protocol for a randomised controlled trial. Trials.

[19] Habr-Gama A, Perez RO, Proscurshim I (2008). Interval between surgery and neoadjuvant chemoradiation therapy for distal rectal cancer: Does delayed surgery have an impact on outcome?. Int J Radiat Oncol Biol Phys.

[20] Garcia-Aguilar J, Smith DD, Avila K, Bergsland EK, Chu P, Krieg RM (2011). Optimal timing of surgery after chemoradiation for advanced rectal cancer: preliminary results of a multicenter, nonrandomized phase II prospective trial. Ann Surg.

[21] Garcia-Aguilar J, Chow OS, Smith DD (2015). Effect of adding mFOLFOX6 after neoadjuvant chemoradiation in locally advanced rectal cancer: a multicentre, phase 2 trial. Lancet Oncol.

[22] Russo AL, Ryan DP, Borger DR (2014). Mutational and clinical predictors of pathologic complete response in the treatment of locally advanced rectal cancer. J Gastrointest Cancer.

[23] Wallin U, Rothenberger D, Lowry A, Luepker R, Mellgren A (2013). CEA – A predictor for pathologic complete response after neoadjuvant therapy for rectal cancer. Dis Colon Rectum.

[24] Jang JK, Choi SH, Park SH (2020). MR tumor regression grade for pathological complete response in rectal cancer post neoadjuvant chemoradiotherapy: a systematic review and meta-analysis for accuracy. Eur Radiol.

[25] Yu SKT, Tait D, Chau I, Brown G (2013). MRI predictive factors for tumor response in rectal cancer following neoadjuvant chemoradiation therapy – implications for induction chemotherapy?. Int J Radiat Oncol Biol Phys.

